# Pleiotropic effects of cancer cells’ secreted factors on human stromal (mesenchymal) stem cells

**DOI:** 10.1186/scrt325

**Published:** 2013-09-17

**Authors:** Mashael Al-toub, Abdulaziz Almusa, Mohammed Almajed, May Al-Nbaheen, Moustapha Kassem, Abdullah Aldahmash, Nehad M Alajez

**Affiliations:** 1 Stem Cell Unit, Department of Anatomy, College of Medicine, King Saud University, Riyadh 11461, Kingdom of Saudi Arabia; 2 Saudi Electronic University, Riyadh, Saudi Arabia; 3 KMEB, Department of Endocrinology, University of Southern Denmark, Odense, Denmark

## Abstract

**Introduction:**

Studying cancer tumors’ microenvironment may reveal a novel role in driving cancer progression and metastasis. The biological interaction between stromal (mesenchymal) stem cells (MSCs) and cancer cells remains incompletely understood. Herein, we investigated the effects of tumor cells’ secreted factors as represented by a panel of human cancer cell lines (breast (MCF7 and MDA-MB-231); prostate (PC-3); lung (NCI-H522); colon (HT-29) and head & neck (FaDu)) on the biological characteristics of MSCs.

**Methods:**

Morphological changes were assessed using fluorescence microscopy. Changes in gene expression were assessed using Agilent microarray and qRT-PCR. GeneSpring 12.1 and DAVID tools were used for bioinformatic and signaling pathway analyses. Cell migration was assessed using a transwell migration system. SB-431542, PF-573228 and PD98059 were used to inhibit transforming growth factor β (TGFβ), focal adhesion kinase (FAK), and mitogen activated protein kinase kinase (MAPKK) pathways, respectively. Interleukin-1β (IL1β) was measured using ELISA.

**Results:**

MSCs exposed to secreted factors present in conditioned media (CM) from FaDu, MDA-MB-231, PC-3 and NCI-H522, but not from MCF7 and HT-29, developed an elongated, spindle-shaped morphology with bipolar processes. In association with phenotypic changes, genome-wide gene expression and bioinformatics analysis revealed an enhanced pro-inflammatory response of those MSCs. Pharmacological inhibitions of FAK and MAPKK severely impaired the pro-inflammatory response of MSCs to tumor CM (approximately 80% to 99%, and 55% to 88% inhibition, respectively), while inhibition of the TGFβ pathway was found to promote the pro-inflammatory response (approximately 3-fold increase). In addition, bioinformatics and pathway analysis of gene expression data from tumor cell lines combined with experimental validation revealed tumor-derived IL1β as one mediator of the pro-inflammatory phenotype observed in MSCs exposed to tumor CM.

MSCs exhibited significant tropism toward secreted factors from the aforementioned tumor cell lines, while both normal and MSCs exposed to tumor CM were capable of attracting human peripheral blood mononuclear cells (PBMCs).

**Conclusions:**

Our data revealed tumor-derived IL1β as one mediator of the pro-inflammatory response in MSCs exposed to tumor CM, which was found to be positively regulated by FAK and MAPK signaling and negatively regulated by TGFβ signaling. Thus, our data support a model where MSCs could promote cancer progression through becoming pro-inflammatory cells within the cancer stroma.

## Introduction

Stromal (mesenchymal) stem cells (MSCs), also referred to as stromal cells, are multipotent cells which are present within the stroma of bone marrow and probably other organs and capable of differentiating into the three canonical lineages: osteoblasts, adipocytes and chondrocytes [1]. Aside from their differentiation potential, MSCs are also capable of migrating to injured tissues and contributing to tissue regeneration [2-4]. Emerging data suggest that MSCs possess immunomodulatory and regenerative properties as they can secrete a large number of growth factors and immune active molecules [5] that can improve tissue survival and suppress the activity of various immune cells, such as alloantigen activated T and B lymphocytes [6,7]. Moreover, MSCs can secrete a large number of paracrine factors, including chemoattractants for endothelial cells, monocytes and macrophages [8]. Several recent studies have reported that bone marrow MSCs migrate to the stromal compartment of tumors [9,10] and that a dynamic interaction between tumor cells and MSCs exists resembling what has been reported during inflammation and, thus, ‘tumors are wounds that never heal’ [11].

Over the past several years, a significant amount of research has emerged documenting a role for MSCs in promoting epithelial-to-mesenchymal transition (ETM) and accelerating tumor growth and metastasis [9,12-14]. In addition, MSCs are being introduced into therapy for a number of clinical indications and there is a concern of possible promoting effects on tumor growth by MSCs [15]. On the other hand, several other studies reported that MSCs exert tumor suppressive effects [16-18]. Therefore, understanding the settings under which MSCs exert promoting versus inhibitory effects on tumor growth and metastasis is currently under intensive investigation.

Given this complex interplay between MSCs and tumor cells, the goal of this study was to assess the cellular and molecular changes in MSCs in response to secreted factors present in conditioned media (CM) from a panel of human tumor cell lines covering a spectrum of human cancers (breast, prostate, lung, colon, and head and neck). Integrated analysis of phenotypic changes, gene expression and bioinformatics revealed a pro-inflammatory response of MSCs when exposed to CM of several tumor cell lines. Interestingly, the biological responses of MSCs were not identical. MSCs responded mainly to tumor cell lines which express high levels of IL1β. We identified tumor-derived IL1β as the prominent cytokine responsible for induction of inflammatory response in MSCs and signaling via focal adhesion kinase (FAK) and, to lesser extent, mitogen activated protein kinase kinase (MAPKK), as key positive regulators of an inflammatory response, while transforming growth factor β (TGFβ) signaling was found to inhibit the response of MSCs to tumor CM. Our data further support a model where MSCs could drive tumorigenicity through induction of inflammation.

## Methods

### Ethics statement

Experiments performed in this study do not need ethics committee approval.

### Cell culture

Tumor cell lines used in this study (breast, MCF7 and MDA-MB-231; prostate, PC-3; lung, NCI-H522; head and neck, FaDu; and colon, HT-29) have been described previously [19-23]. The human telomerized hMSC-TERT-GFP cell line was developed by Dr Kassem, Odense, Denmark [24,25]. All cell lines were maintained in (D)MEM 4.5g/L glucose (Invitrogen Corp., Carlsbad, CA, USA) and supplemented with 10% fetal bovine serum, 1% NEAA, 1% L-glutamine, 100 mg/L penicillin and 100 mg/L streptomycin at 37°C and 5% CO_2._ For TGFβ inhibition experiments, MSC were cultured as described above and were exposed to MDA-MB-231 CM in the presence of 10 μM SB-431542 (Sigma, St. Louis, MO, USA). Control wells were treated with dimethyl sulfoxide (DMSO). CM plus SB-431542 or vehicle (DMSO, Sigma) was changed every three to four days for the duration of the experiment. Recombinant human IL1β and IL6 were purchased from Invitrogen. FAK inhibitor (PF-573228) and mitogen activated protein kinase kinase (MAPKK) inhibitor (PD98059) were purchase from Sigma and were reconstituted in DMSO.

### Collection of tumor cell lines conditioned media

The tumor cell lines, MCF7, HT-29, MDA-MB-231, PC-3, NCI-H522 and FaDu were seeded in six-well plates at 1 × 10^6^/well (4 ml total) in (D)MEM supplemented with 10% fetal bovine serum (FBS), 1% NEAA and 1% penicillin/streptomycin and incubated at 37°C and 5% CO_2_. Forty-eight hours later (cells were approximately 90% confluent), CM from the tumor cell lines were collected and spun down at 300 × g for 10 minutes to remove any cellular content and debris. In some experiments, CM was passed through a 0.45 μΜ filter to remove any remaining cellular content and debris. The hMSC-TERT-GFP cells were then seeded in 24-well plates at 8 × 10^4^/ml in the collected CM (80% tumor CM + 20% fresh medium). The MSCs were exposed to fresh CM every two to three days for the duration of the experiment.

### Quantification of secreted IL1β using ELISA

Quantification of secreted IL1β from tumor cell lines or from MSCs exposed to tumor CM was done using the LEGEND MAX™ Human IL-1β ELISA Kit (Biolegened Inc., San Diego, CA, USA) according to the manufacturer’s recommendations. CM from tumor cell lines were collected as described above and stored at −80°C for the ELISA. To measure secreted IL1β from control MSCs or MSCs exposed to tumor CM, MSCs were exposed to MCF7 or FaDu CM for seven days. Subsequently, the cells were washed three times with PBS and fresh culture medium was added. CM was collected for the ELISA 72 hours later.

### Fluorescence microscopy

Microscopy was performed on the indicated days using a Nikon**®** ECLIPSE Ti-U inverted fluorescence microscope. Cells were either imaged directly or were washed with 1x PBS, followed by staining with Hoechst 33342 (10 μg/ml) in PBS for 10 minutes at 37°C.

### Microarray experiment

Human MSCs were exposed to FaDu tumor CM as described above. On day 7, when the spindle-shape phenotype was usually observed, the cells from three different replicates were harvested and RNA was extracted using the Roche MagNA Pure automated nucleic acid purification system (Roche Diagnostics GmbH, Mannheim, Germany). RNA quantity and quality were measured using the NanoDrop 2000 spectrophotometer (Thermo Scientific, Wilmington, DE, USA). Control RNA was collected from the same batch of MSCs exposed to normal medium. Extracted RNA was labeled and then hybridized to the Agilent Human GE 4x44K v2 Microarray chip (Agilent Technologies, Santa Carla, CA, USA). All microarray experiments were conducted at the Microarray Core Facility (Stem Cell Unit, King Saud University College of Medicine, Riyadh, Saudi Arabia). Data analyses were conducted using GeneSpring X software (Agilent Technologies) and the DAVID bioinformatic tool as described previously [26]. Microarray data were deposited in the Gene Expression Omnibus (GEO) database (accession number GSE50722).

### Quantitative real-time polymerase chain reaction

The expression of a panel of genes identified from the microarray experiment in MSCs exposed to tumor CM from FaDu, MCF7, MDA-MB-231, PC-3 and NCI-H522 was performed using the StepOne Plus PCR system (Applied Biosystems Inc, Foster City, CA, USA); the primers used are listed in Table 1. Briefly, RNA was extracted using the Roche MagNA Pure automated nucleic acid purification system (Roche Diagnostics GmbH). cDNA was generated using a High-Capacity cDNA Reverse Transcription Kit (Applied Biosystems Inc). The real-time PCR reaction was run using Fast SYBR® Green Master Mix (Applied Biosystems Inc). The relative fold change in RNA expression was calculated using the 2^−ΔΔ*C*t^ method, where the average of Δ*C*t values for the amplicon of interest were normalized to that of an endogenous gene (GAPDH), compared with control specimens [27].

**Table 1:** Primer sequences used for qRT-PCR

**No.**	**Name**	**Sequence**
**1**	**CCL3**	
	**F**	5’ AAGGACACGGGCAGCAGACA 3’
	**R**	5’ AGCAGCAAGTGATGCAGAGAACTGG 3’
**2**	**CCL5**	
	**F**	5’ TACATTGCCCGCCCACTGCC 3’
	**R**	5’ TCGGGTGACAAAGACGACTGCT 3’
**3**	**CCL8**	
	**F**	5’ GGGACTTGCTCAGCCAGATTCAGT 3’
	**R**	5’ CAGCACAGACCTCCTTGCCCC 3’
**4**	**CXCL2**	
	**F**	5’ GGGGTTCGCCGTTCTCGGA 3’
	**R**	5’ TGCGAGGAGGAGAGCTGGCAA 3’
**5**	**CXCL3**	
	**F**	5’ CGCCCAAACCGAAGTCATAGCCA 3’
	**R**	5’ TGGTAAGGGCAGGGACCACCC 3’
**6**	**CXCL5**	
	**F**	5’ GTTGAGAGAGCTGCGTTGCGT 3’
	**R**	5’ TCAGGGAGGCTACCACTTCCACC 3’
**7**	**CXCL6**	
	**F**	5’ GGTAAACTGCAGGTGTTCCCCGC 3’
	**R**	5’ CCCGTTCTTCAGGGAGGCTACCA 3’
**8**	**IL6**	
	**F**	5’ CGAGCCCACCGGGAACGAAA 3’
	**R**	5’ GGACCGAAGGCGCTTGTGGAG 3’
**9**	**IL1B**	
	**F**	5’ AGGCACAAGGCACAACAGGCT 3’
	**R**	5’ TGGCTGCTTCAGACACTTGAGCAAT 3’
**10**	**IGF2**	
	**F**	5’ GCTCTGCCCCGTCGCACATT 3’
	**R**	5’ TTGGTGTCTGGAAGCCGGCGA 3’
**11**	**EHF**	
	**F**	5’ GGCATGGGGTTGCCGGAGAG 3’
	**R**	5’ CTGGAAACATTGCACGTGGAGTAGC 3’
**12**	**CSF2**	
	**F**	5’ GACCTCCAGGAGCCGACCTGC 3’
	**R**	5’ AGTTTCCGGGGTTGGAGGGCA 3’
**13**	**SAA1**	
	**F**	5’ GGCTTTTGATGGGGCTCGGGA 3’
	**R**	5’ CCCCCAGGTCCCCTTTTGGC 3’
**14**	**MMP12**	
	**F**	5’ TGCCCGTGGAGCTCATGGAGAC 3’
	**R**	5’ TGTGCATCCCCTCCAATGCCAG 3’

### *In vitro* angiogenesis assay

An *in vitro* angiogenesis assay was conducted as we described previously [28]. MSCs were seeded in a 24-well plate at 8 × 10^4^/well in normal or CM from FaDu or MDA-MB-231 cell lines. On day 10, a 24-well plate was prepared for the matrigel assay by adding 250 μl of chilled Matrigel**®** (10 mg/mL, Basement Membrane Matrix, BD Biosciences, San Diego, CA, USA) for each well, and then the plate was incubated at 37°C for 30 minutes. MSCs exposed to CM or control were trypsinized and cultured in 24-well plates pre-coated with Matrigel**®** at 1 × 10^5^ in 500 μl of media. Images were taken at 2 hours and 72 hours using a Nikon**®** ECLIPSE Ti-U inverted fluorescence microscope.

### Adipogenic and osteoblastic differentiation

MSCs were seeded in a 24-well plate at 8 × 10^4^/well in normal or CM from FaDu or MDA-MB-231 cell lines. On day 10, cells were switched to adipogenic ((D)MEM supplemented with 10% FBS, 10% horse serum (Sigma), 1% penicillin/streptomycin, 100 nM dexamethasone, 0.45 mM isobutyl methyl xanthine ((IBMX) (Sigma)), 3 μg/mL insulin (Sigma) and 1 μM rosiglitazone ((BRL49653) (Novo Nordisk, Bagsvaerd, Denmark)) or osteogenic ((D)MEM containing 10% FBS, 1% penicillin/streptomycin, 50 μg/mL L-ascorbic acid (Wako Chemicals GmbH, Neuss, Germany), 10 mM β-glycerophosphate (Sigma), and 10 nM calcitriol ((1α,25-dihydroxy vitamin D3) (Sigma)), 10 nM dexamethasone (Sigma)) differentiation medium as we previously described [28]. Medium was changed every three days. On day 6, adipocytic and osteoblastic differentiation was measured using Oil-Red-O and alkaline phosphatase (ALP) staining, respectively.

### Transwell cell migration assay

On the day of the experiment, tumor cells were trypsinized and counted using an automated cell counter (Vi-Cell XR cell viability analyzer, Beckman Coulter Inc, Fullerton, CA, USA). Subsequently, 4 × 10^5^ cells were seeded in 2 ml of low serum (D)MEM ((D)MEM + 1% FBS, 1% NEAA, 1% penicillin/streptomycin) in the lower chamber of a 12-well transwell migration system (BD Biosciences). Twenty four hours later, 1 × 10^5^ hMSC were re-suspended in 1 ml of low serum (D)MEM in the upper chamber. MSC migration toward (D)MEM supplemented with 1% FBS was used as a negative control. Twenty four hours later, inserts were removed, and cells on the upper surface were scraped using a cotton swap, and, subsequently, were fixed with 4% Paraformaldehyde (PFA) for 20 minutes, followed by H & E staining. Stained inserts were subsequently cut and mounted on microscope slides. Digital slides were taken using a digital microscope and eight (1600 × 1000 mcM2) fields were counted from each insert. For leukocyte migration, MSCs were exposed to tumor CM for seven days. Subsequently, wells were washed and fresh (D)MEM + 0.5% BSA was added. CM from control MSCs ((D)MEM + 0.5% BSA) or MSCs exposed to FaDu CM ((D)MEM + 0.5% BSA) was collected 72 hours later and used in the migration experiment. Human peripheral blood mononuclear cells (PBMCs) (1 × 10^5^) were seeded in the upper chamber, while control medium or MSC CM was placed in the lower chamber. Two hours later, images of migrating cells were taken using a Zeiss inverted microscope.

### Statistical analysis

Statistical analyses and graphing were performed using Microsoft**®** excel 2007 and Graphpad Prism 6.0 software (Graphpad**®** software, San Diego, CA, USA). *P* values were calculated using the two-tailed *t*-test. Correlative analyses were done using Pearson’s correlation using Graphpad prism 6.0.

## Results

### Effects of conditioned media on MSCs morphology and gene expression

Initially, we assessed the effect of CM from a FaDu tumor cell line on MSC morphology. We observed a striking difference in the shape of MSCs following five to seven days exposure to FaDu CM compared to control MSC culture (* 1a). MSCs exposed to FaDu CM exhibited a spindle-shaped morphology and were more elongated with bipolar processes compared to the larger control MSCs with flattened morphology.

**Figure 1:**
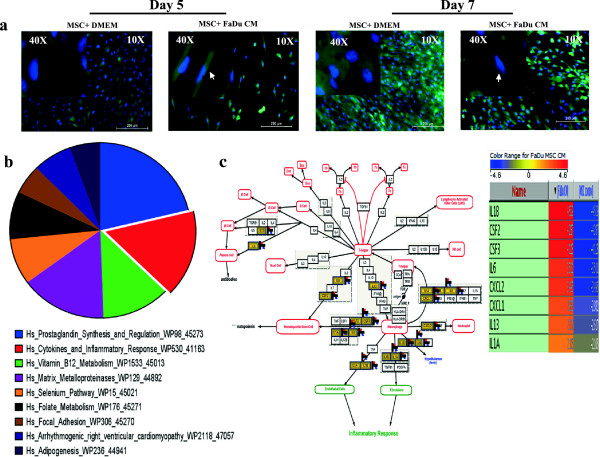
**Effects of FaDu conditioned medium (CM) on human MSC morphology and gene expression. (a)** Representative micrographs of MSC-GFP cells grown under normal conditions (left panel) or exposed to FaDu CM (right panel). Hoechst 33342 was used for nuclear staining and images were obtained at the indicated time points (10× magnification, 200 μm scale bar). Arrow heads point to MSCs with fibroblastic morphology in CM treated cells. **(b)** MSCs grown under normal conditions or exposed to FaDu CM were subjected to microarray analysis. Differentially upregulated genes in MSCs exposed to FaDu CM were subsequently subjected to pathway analysis as described in Methods. The pie chart represents the top ten pathways where the pie size represents percent enrichment of the pathway. **(c)** Genes in the cytokine and inflammatory response pathway were among the most highly enriched category in the microarray data. MSCs, mesenchymal stem cells.

This striking finding led us to hypothesize that secreted factors from FaDu tumor cells mediated biological changes in MSC phenotype and gene expression. To identifiy those genetic changes, we conducted global gene expression analysis of MSCs exposed to FaDu CM compared to control MSCs cultures. Microarray data and pathway analyses of the upregulated genes revealed significant enrichment for genes involved in inflammatory response-related cytokines and chemokines, for example, IL1β, CSF2, CSF3, IL6, CXCL2, CXCL1, IL13 and IL1α, as well as metalloproteinases (Figure 1b, c, and Additional file 1: Table S1).

### Effects of CM from tumor cell lines on MSC morphology and gene expression is cell line-dependent

**Figure 2:**
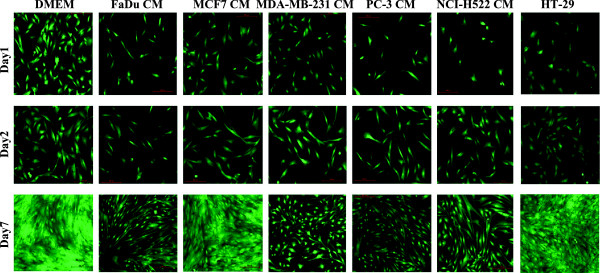
**Comparative analysis of morphological changes in MSCs exposed to conditioned medium (CM) from a panel of human cancer cell lines.** MSCs were grown under normal conditions ((D)MEM) or were exposed to CM from the indicated cancer cell lines (FaDu, MCF7, MDA-MB-231, PC-3, NCI-H522 and HT-29) and, subsequently, images were obtained on days1, 2 and 7. Representative micrographs from at least three independent experiments are shown. All images were taken using 10x magnification. MSCs, mesenchymal stem cells.

We subsequently sought to determine if secreted factors from other tumor cell lines exert similar phenotypic and gene expression changes on MSCs to those seen with FaDu. MSCs were exposed to CM collected from a panel of human cancer cell lines (MCF7 and MDA-MB-231 (breast), PC-3 (prostate), NCI-H522 (lung) and HT-29 (colon)). Changes in morphology were evaluated on days 1, 2, and 7. Interestingly, MSCs exposed to all cell lines, except MCF7 and HT-29 CM, exhibited marked changes in appearance compared to control cells (Figure 2). MSCs exposed to PC-3 developed spindle shape morphology, with bipolar cellular projections at day 7 and MSCs exposed to NCI-H522 and MDA-MB-231 CM exhibited similar morphological changes but were less pronounced. Interestingly, these morphological changes were absent in MSC cultures exposed to MCF7 and HT-29 CM. Nonetheless, the confluency of MSCs was relatively higher in control, MCF7 and HT-29 CM compared to that in FaDu, MDA-MB-231, PC-3 and NCI-H522 CM, suggesting a possible growth inhibitory effect of the latter CM on MSC growth. In fact, MSCs exposed to FaDu CM had a relatively slower growth rate compared to control MSCs, which was also associated with a decrease in the G1 and increase in the G2M phase of the cell cycle [see Additional file 2: Figure S1].

**Figure 3:**
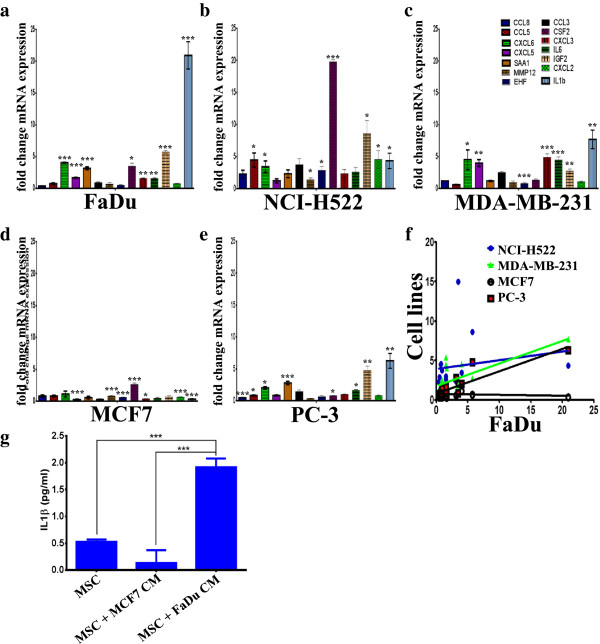
**Validation of selected genes in the cytokine, inflammation, and metalloproteases pathways in MSCs exposed to conditioned medium (CM) from a panel of human cancer cell lines.** MSCs were exposed to CM as described in Figure 2, then qRT-PCR was utilized to validate the expression of selected genes in the cytokine, inflammation, and metalloproteases pathways in MSCs exposed to CM of FaDu **(a)**, NCI-H522 **(b)**, MDA-MB-231 **(c)**, MCF7 **(d)** and PC-3 **(e)**. Data are presented as mean fold change (relative to control MSCs) ± S.D. from at least two independent experiments, n = 4. *P* values * <0.05, ** <0.005, *** <0.0005. **(f)** Correlative analysis of the expression of the aforementioned genes in FaDu versus the other cell lines. **(g)** Quantification of secreted IL1β by ELISA from MSCs exposed to MCF7 or FaDu CM. Data are presented as mean ± S.D. n = 3. MSCs, mesenchymal stem cells.

Given our finding that the highest enrichment in upregulated genes in MSCs exposed to FaDu CM was in the category of inflammatory cytokines and matrix metalloproteinases (MMPs) (Figure 1b, c, and Additional file 1: Table S1 and Additional file 3: Table S2), the expression of a selected group of genes in MSCs exposed to FaDu, in addition to the CM from other cancer cell lines was subsequently validated using qRT-PCR. Overall, our data revealed similar expression patterns of the selected genes in MSCs exposed to FaDu, NCI-H522, MDA-MB-231 and PC-3 CM, while the expression of those genes was lower in MSCs exposed to MCF7 CM (Figure 3a-e). In addition, we found a significant correlation between the expression of these genes in MSCs exposed to FaDu, MDA-MB-231 and PC-3 CM, but not in MSCs exposed to MCF7 CM (Figure 3f). As seen in Figure 2, the gene expression data correlated with the observed phenotypic changes. MSCs exposed to FaDu CM secreted a significant amount of IL1β, compared to control MSCs or MSCs exposed to MCF7 CM (Figure 3g), which is concordant with the qRT-PCR data.

### Pro-inflammatory response of MSCs exposed to FaDu CM is mediated mainly through focal adhesion kinase signaling

**Figure 4:**
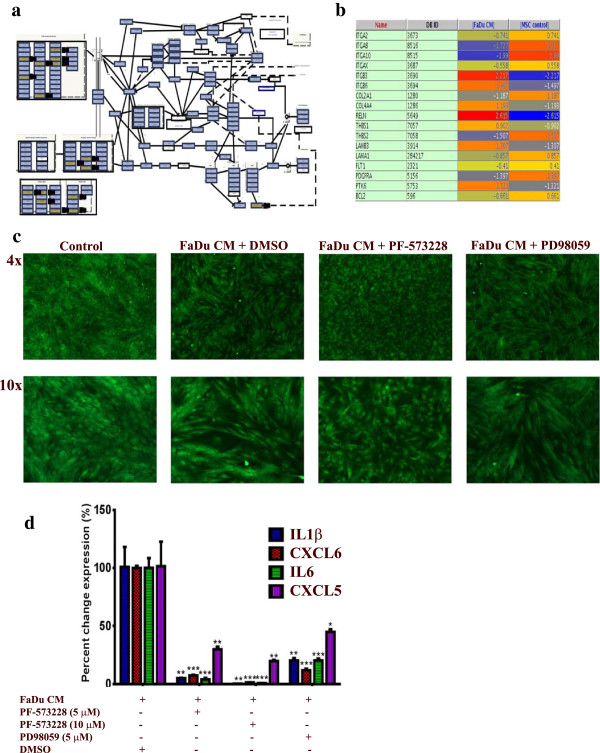
**Inhibition of FAK and MAPK abrogates the pro-inflammatory response in MSCs exposed to FaDU CM. (a)** The focal adhesion kinase (FAK) pathway was among the top upregulated pathways in MSCs exposed to FaDu CM. **(b)** The list of differentially expressed genes in the FAK pathway in MSCs exposed to FaDu CM. **(c)** Pharmacological inhibition of FAK (5 μM, PF-573228, Sigma) or MAPKK (5 μM, PD98059, Sigma) led to significant inhibition of the pro-inflammatory response in MSCs exposed to FaDu CM. **(d)** Quantification of a representative set of genes in the cytokine and inflammatory response pathway in MSCs exposed to FaDu CM in the presence of DMSO, FAK, or MAPKK inhibitors. Data are presented as percent change in gene expression relative to MSCs exposed to FaDu CM + DMSO. Data are presented as mean ± S.D. n = 3. CM, conditioned media; DMSO, dimethyl sulfoxide, FAK, focal adhesion kinase; MAPKK, mitogen activated protein kinase kinase; MSCs, mesenchymal stem cells.

Pathway analysis of differentially expressed genes in MSCs exposed to FaDu CM revealed multiple enriched pathways. Among those, FAK (*P* = 2.1 × 10 ^-5^) and, to lesser extent, MAPK (*P* = 0.03) were very prominent [see Additional file 1: Table S1]. Differentially expressed genes in the FAK pathway are shown in Figure 4a and b. To assess whether FAK and MAPK pathways are indeed involved in regulating the pro-inflammatory response of MSCs exposed to tumor CM, MSCs were exposed to control or FaDu CM in the presence of PF-573228 (FAK inhibitor), PD98059 (MAPKK inhibitor) or DMSO. On day 5, cells were monitored for phenotypic changes. As shown in Figure 4c, FAK inhibitor almost completely inhibited the pro-inflammatory phenotype, while MAPKK inhibitor had a less pronounced effect. qRT-PCR analysis of a panel of pro-inflammatory cytokines (IL1β, CXCL6, IL6 and CXCL5) revealed drastic inhibition of the expression of those cytokines in the presence of FAK inhibitor in a dose dependent manner (Figure 4d). MAPKK inhibitor also significantly inhibited the pro-inflammatory response in MSCs exposed to FaDu CM, but less than that seen with the FAK inhibitor (Figure 4d).

### Signaling via TGFβ negatively regulates the pro-inflammatory response of MSCs to FaDu CM

**Figure 5:**
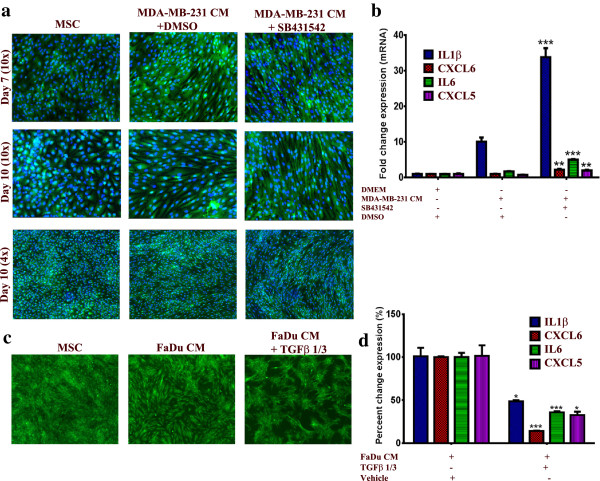
**TGFβ signaling negatively regulates the pro-inflammatory response of MSCs exposed to tumor CM. (a)** MSCs were cultured as described in Methods and then were exposed to MDA-MB-231 CM in the presence of 10 μM SB-431542 or DMSO. On the indicated days, nuclei were stained with Hoechst 33342 and cells were visualized under a florescent microscope. Data are representative of at least three independent experiments. **(b)** Quantification of a representative set of genes in the cytokine and inflammatory response pathway in MSCs exposed to MDA-MB-231 CM in the presence of 10 μM SB-431542 or DMSO from **a**. Data are presented as the fold change in gene expression relative to control MSCs. Data are presented as mean ± S.D. n = 3. **(c)** MSCs were cultured as described in Methods and then were exposed to FaDu CM in the presence of 10 μg/ml TGFβ1 and TGFβ3. On Day 5, cells were visualized under a florescent microscope (4x). **(d)** Quantification of a representative set of genes in the cytokine and inflammatory response pathway in MSCs exposed to FaDu CM in the presence of 10 μg/ml TGFβ1 and TGFβ3 from **c**. Data are presented as percent change in gene expression relative to MSCs exposed to FaDu CM + vehicle (dH_2_O). Data are presented as mean ± S.D. n = 3. CM, conditioned media; DMSO, dimethyl sulfoxide; MSCs, mesenchymal stem cells.

Given its critical role in tumorigenicity and in regulating the differentiation of MSCs [29-31], we hypothesized that changes in TGFβ signaling could potentially regulate the observed changes in the phenotype of MSCs. Interestingly, pharmacological inhibition of the TGFβ receptor kinase using SB-431542 (10 μM) in MSCs in the presence of MDA-MB-231 CM (this cell line was selected because it has the highest expression of TGFβ among all cell lines used in this study, data not shown) led to significant enhancement in the characteristic morphology of MSCs (Figure 5a). Concordant with that, the expression of the pro-inflammatory cytokine panel was significantly increased in the presence of SB-431542 compared to control DMSO (>3-fold), Figure 5b. On the other hand, treating MSCs with recombinant TGFβ1 and TGFβ3 in the presence of FaDu CM (this cell line was selected for this experiment since it induced the strongest phenotype and has low TGFβ expression compared to MDA-MB-231) led to significant inhibition of the pro-inflammatory phenotype at the cellular and molecular levels (Figure 5c and d). Therefore, our data indicate an inhibitory role for TGFβ signaling on mediating the observed changes in the MSCs phenotype.

### MSCs exposed to tumor CM have diminished multilineage differentiation potential

**Figure 6:**
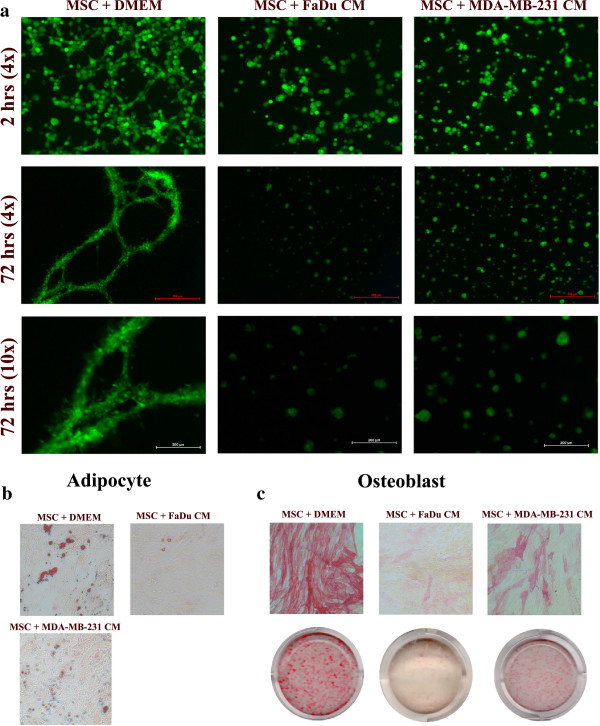
**MSCs exposed to tumor CM lose their multipotent differentiation potential. (a)** Control MSCs or MSCs exposed to FaDu or MDA-MB-231 CM (10 days) were harvested and seeded on top of Matrigel**®** as indicated in Methods sections. Vessel-like tubular formation was assessed at 2 hours and 72 hours using a fluorescence microscope at 4x and 10x, as indicated. Data are representative of at least two experiments. Control MSCs or MSCs exposed to FaDu or MDA-MB-231 CM (10 days) were switched to adipogenic **(b)** or osteogenic induction media **(c)**. On day 6, adipocyte differentiation was measured using Oil-Red-O staining **(b)**, while osteoblast differentiation was measured using alkaline phosphatase staining (ALP) **(c)**. Data are representative of at least two experiments. CM, conditioned media; MSCs, mesenchymal stem cells.

Recent study using an *in vitro* angiogenesis assay has indicated that human MSCs exposed to CM from a glioblastoma cell line form a vascular-like tubular network [32]. Therefore, MSCs were exposed to CM from two selected cancer cell lines: FaDu and MDA-MB-231 for 10 days, then cells were seeded on a Matrigel**®** matrix and their ability to form a vascular-like tubular network was assessed during a 72-hour period. Control MSCs began to align and form tubular network structures as early as two hours post-cultivation on Matrigel**®** (Figure 6a, upper left), which was very noticeable by 72 hours (Figure 6a, middle and bottom left). MSCs exposed to FaDu and MDA-MB-231 CM failed to form any tubular structures up to 72 hours (Figure 6a, middle and right panels). Similarly, MSCs exposed to FaDu or MDA-MB-231 CM had diminished adipogenic and osteogenic differentiation potential (Figure 6b and c). Interestingly, the inhibitory effect was more evident in MSCs exposed to FaDu CM compared to MDA-MB-231 CM, which seems to correlate with the induction of a pro-inflammatory response in MSCs (compare Figure 6b, c, and Figure 3a, c). Taken together, these data suggest that exposing MSCs to FaDu or MDA-MB-231 CM induced the differentiation of MSCs into pro-inflammatory cells, which was also associated with diminished multilineage differentiation potential.

### Clustering analysis of tumor cell lines gene expression profile

**Figure 7:**
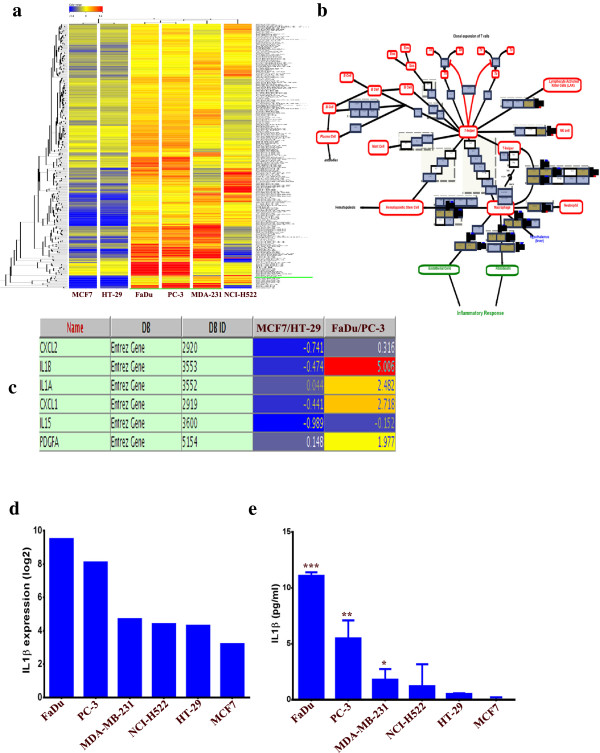
**Cluster and pathway analysis of basal gene expression in FaDu, NCI-H522, MDA-MB-231, MCF7, PC-3 and HT-29 tumor cell lines. (a)** Clustering analysis of the tumor cell lines indicated close clustering for FaDu and PC-3, followed by MDA-MB-231 and NCI-H522, while MCF7 and HT-29 did not cluster readily with the group. Clustering analyses were performed on differentially expressed genes in FaDu and PC-3 relative to MCF7 and HT-29. **(b)** Cytokine and inflammatory response pathway was among the top enriched pathways in differentially expressed genes between FaDu and PC-3 relative to MCF7 and HT-29. **(c)** Genes in the cytokine and inflammatory response pathways from **b** and their expression levels are shown. **(d)** mRNA expression level of IL1β in different tumor cell lines from the microarray data. **(e)** Quantification of secreted IL1β by ELISA from different tumor CM. Data are presented as mean ± S.D. n = 3. CM, conditioned media.

We subsequently determined if the changes in MSCs phenotype and gene expression pattern post exposure to tumor CM are associated with the genetic characteristics of the tumor cell lines employed. Thus, publicly available gene expression data for FaDu, MCF7, HT-29, MDA-MB-231, NCI-H522 and PC-3 were retrieved from The Gene Expression Omnibus ( [33]; Series Accession GSE36133) and were subjected to bioinformatics. Since the pro-inflammatory phenotype was most evident in MSCs exposed to FaDu and PC-3 CM, while it was absent in MSCs exposed to MCF7 or HT-29 CM, we performed clustering analyses on the significantly differentially expressed genes in FaDu and PC-3, compared to MCF7 and HT-29 cell lines using GeneSpring X software. Data presented in Figure 7a revealed close clustering of the FaDu and PC-3, followed by MDA-MB-231 and NCI-H522, while MCF7 and HT-29 exhibited poor clustering with the above mentioned cell lines. Interestingly, the cytokine and inflammatory response was among the top upregulated pathways in the differentially expressed genes in FaDu and PC-3, compared to MCF7 and HT-29 (Figure 7b and c). IL1β was the most highly upregulated gene in FaDu and PC-3 compared to MCF7 and HT-29 (Figure 7c, and d). Concordant with that, FaDu and PC-3 secreted the largest amount of IL1β, followed by MDA-MB-231 and NCI-H522, while HT-29 and MCF7 secreted the smallest amount of IL1β. Interestingly, IL1β production by tumor cells seemed to correlate with the induced pro-inflammatory phenotype (compare Figure 7e and Figure 3).

### IL1β treatment induced a pro-inflammatory phenotype in MSCs similar to that induced by tumor CM

**Figure 8:**
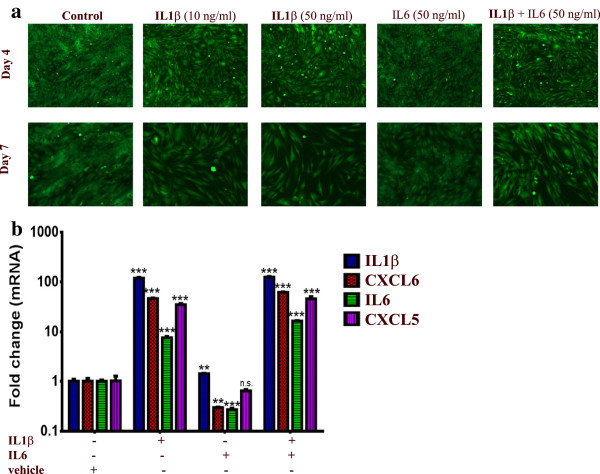
**IL1β treatment induced a pro-inflammatory response in MSCs. (a)** MSCs were cultured in normal (D)MEM in the presence of recombinant IL1β (10 and 50 ng/ml), recombinant IL6 (50 ng/ml), both IL1β and IL6 (50 ng/ml each) or in the presence of vehicle control (dH_2_O). Images were taken on day 4 and 7 (4x). **(b)** Quantification of a representative set of genes in the cytokine and inflammatory response pathway in MSCs exposed to different cytokines from **(a)**. Data are presented as fold change in gene expression relative to MSCs exposed to vehicle. Data are presented as mean ± S.D., n = 3. MSCs, mesenchymal stem cells.

Data presented in Figure 7 suggest that tumor derived IL1β might be the main cytokine responsible for the pro-inflammatory response in MSCs exposed to tumor CM. To test this hypothesis, MSCs were grown in normal (D)MEM in the presence of IL1β or IL6. Interestingly, treating MSCs with IL1β phenocopied the pro-inflammatory phenotype seen in MSCs exposed to tumor CM, while IL6 treatment had almost no effect on the MSC phenotype (Figure 8a). The effect of IL1β was dose-dependent [see Additional file 4: Figure S2]. Similarly, exposing MSCs to IL1β led to significant upregulation of the pro-inflammatory cytokines (7.5 to 120 fold). On the other hand, exposing MSCs to IL6 had only slight increase in IL1β expression (1.4 fold, Figure 8b).

### MSCs exhibited significant tropism toward different tumor cell lines *in vitro*

**Figure 9:**
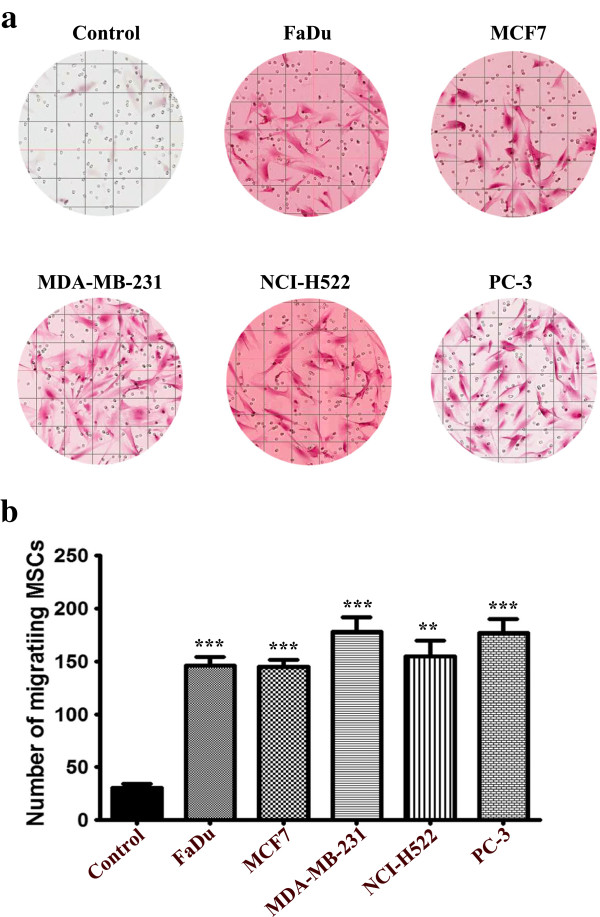
**Tumor cells are capable of attracting human MSCs *in vitro*.** Tumor CM ((D)MEM + 1% FBS) was placed in the lower chamber of a transwell migration system, while MSCs were seeded in the upper chamber. Twenty-four hours later, the number of migrating MSCs was evaluated. **(a)** H & E staining of the cells migrating toward CM from the indicated tumor cell lines. Migration toward (D)MEM + 1% FBS was used as baseline control. **(b)** Slides were scanned and the number of migrating cells in eight (1,600 x 1,000 mcM2) fields was counted. Data are presented as mean ± S.D, n = 8. *P* value * <0.05, ** <0.005, *** <0.0005. CM, conditioned media; MSCs, mesenchymal stem cells.

To establish a model of a potential crosstalk between MSCs and tumor cells, we then determined if different tumor cell lines are chemoattractant to MSCs *in vitro*. To that end, we conducted a transwell migration experiment in which different tumor cell lines were seeded in the lower chamber under low serum conditions, while MSCs were seeded in the upper chamber. Data presented in Figure 9a revealed a significant increase in MSC migration toward all tumor cell lines compared to control media, thus confirming the potential tropism of MSCs toward secreted factors from the tumor cells. Although all tumor cell lines exhibited similar capability to attract MSCs, the highest migration was seen toward MDA-MB-231 and PC-3 cell lines (Figure 9b).

### Control MSCs or MSCs exposed to tumor CM are capable of attracting human PBMCs

**Figure 10:**
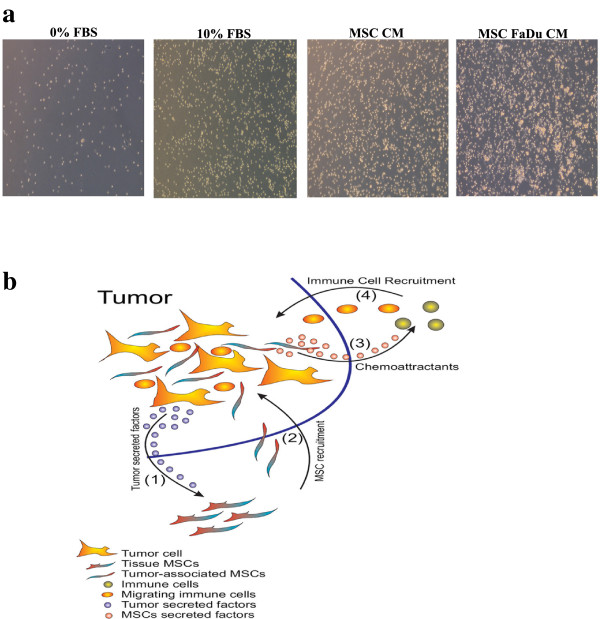
**Both control and MSCs exposed to FaDu conditioned medium (CM) are capable of attracting human PBMCs. (a)** Conditioned medium from MSCs or MSCs exposed to FaDu CM were collected and placed in the lower chamber of a transwell migration system, while 1 x 10^5^ PBMCs were placed in the upper chamber. Representative images of PBMCs migrating to the lower chamber are shown. Data are representative of at least two independent experiments, conducted in duplicate. **(b)** A model depicting the crosstalk between tumor cells, MSCs and immune cells. (1) Tumor cells secrete soluble factors which attract MSCs (2). MSCs at the tumor site become tumor-associated MSCs with enhanced inflammatory responses and secreted chemokines (3) which attract immune cells (4) to the tumor site, collectively acting to drive tumorigenicity via enhanced inflammation as one potential mechanism of tumor progression. CM, conditioned media; MSCs, mesenchymal stem cells; PBMCs, peripheral blood mononuclear cells.

Previous studies have indicated a role for tumor infiltrating immune cells in contributing to inflammation, thus promoting tumorigenicity [34,35]. Therefore, we investigated whether human MSCs or MSCs exposed to FaDu CM are capable of attracting human PBMCs. CM ((D)MEM + 0.5% BSA) from MSCs or MSCs exposed to FaDu CM were collected and placed in the lower chamber in a transwell migration system, while 1 × 10^5^ human PBMCs were seeded in the upper chamber. As shown in Figure 10a, a significant increase in PBMC migration toward MSCs or MSCs exposed to FaDu CM was observed.

## Discussion

For several decades, the molecular changes within tumor cells were studied in order to understand factors responsible for promoting tumor progression and metastasis, while little attention was paid to the possible contributory role of tumor microenvironment. Recent evidence suggests that the tumor microenvironment, which is composed of a very complex network of extracellular matrix (ECM) proteins and many cell types, such as endothelial cells, stromal (mesenchymal) stem cells, pericytes, fibroblasts and immune cells, plays a critical role in tumor progression and metastasis [36,37]. Among these components, MSCs have been the focus of intensive investigation [9,17,38-45].

In the present report, we examined the crosstalk between tumor cells and MSCs and we investigated the effect(s) of tumor secreted factors on MSCs at the cellular and molecular levels. As surrogates for malignant tumors, we employed a number of well characterized cancer cell lines. We reported that secreted factors from FaDu cells led to significant morphological and genetic changes in MSCs with enhanced expression of pro-inflammatory cytokines, and similar responses were also observed when additional tumor cell lines were evaluated. However, these effects were not universal for all malignant cell lines. For example, MCF7 and HT-29 did not exert these effects. Our findings corroborate recent findings of the presence of morphological and functional changes in mouse MSCs in response to cancer cell lines (MDA-MB-231, PANC-1 and U87) CM [46], which exhibit a carcinoma-associated fibroblast (CAF)-like myofibroblastic phenotype.

Interestingly, several of the pro-inflammatory molecules identified in the current study have been linked to cancer progression. For instance, cancer cells that overexpress CXCL1 and 2 were found to be more primed for survival at metastatic sites, and are capable of attracting CD11b(+)Gr1(+) myeloid cells into the tumor that enhance cancer cell survival and enhance their chemoresistance and metastatic ability [47]. In addition to that, CXCL2 was also found to be involved in cancer-associated bone destruction [48]. A recent study has reported differentiation of human MSCs into pericyte–like cells upon exposure to glioblastoma tumor CM [32]. In our current study, we observed no evidence of differentiation of MSCs into pericytes or endothelial-like cells using an *in vitro* angiogenesis assay (Figure 6a). In fact, MSCs exposed to FaDu or MDA-MB-231 CM failed to form any vascular-like tubular networks compared to control MSCs, suggesting MSCs have lost their ability to support angiogenesis [49]. Nonetheless, MSCs exposed to tumor CM also exhibited poor adipocytic and osteoblastic differentiation potential (Figure 6b), probably as a result of differentiation into pro-inflammatory cells. Glioblastoma are known for their high angiogenic capability and the secretion of high levels of VEGF [50], which might account for the variable effects of CM from breast, lung, prostate, and head and neck cancer models investigated in the current study compared to published glioblastoma data [32]; hence, the response of MSCs to tumor secreted factors can vary depending on the tumor type.

Our gene expression data revealed significant correlation between the expression of a panel of genes involved in inflammation and the metalloprotease pathway (CCL8, CCL5, CXCL6, CXCL5, SAA1, MMP12, EHF, CCL3, CSF2, CXCL3, IL6, IGF2, CXCL2 and IL1b) in MSCs exposed to FaDu and to those exposed to MDA-MB-231, PC-3 and NCI-522 CM, while the expression of these genes was almost unchanged in MSCs exposed to MCF7 CM (Figure 3). These data support our hypothesis of the ability of tumor cells to recruit MSCs to their stroma and which in turn induce inflammation, either directly or through recruiting circulating immune cells (Figure 10b). It seems that this model does not apply to all cancer models since in the MCF7 model, MSCs seemed to promote tumorigenicity via direct interaction with tumor cells (Al-toub *et al*., in preparation).

Bioinformatics and pathway analysis of gene expression data from tumor cell lines revealed that the phenotypic changes were mostly observed in MSCs exposed to CM from cell lines with a pro-inflammatory nature (such as, FaDu and PC-3, Figure 7c). Indeed our investigation has identified tumor-derived IL1β to be the primary driver of the pro-inflammatory phenotype observed in MSCs exposed to tumor CM, whereas treating MSCs with recombinant IL1β mimicked the effects of tumor CM at the cellular and molecular level (Figures 7d-e and 8a-b).

Nonetheless, we also identified signaling via FAK and, to lesser extent, MAPK to be critical for the induction of the observed phenotype (Figure 4). In contrast, pharmacological inhibition of TGFβ signaling in MSCs led to substantial enhancement in the observed changes in phenotype and gene expression in MSCs exposed to MDA-MB-231 CM (Figure 5a and b), which was also associated with a slight increase in cell proliferation [see Additional file 5: Figure S3]. Treating MSCs with recombinant TGFβ1 and TGFβ3 in the presence of FaDu CM led to significant inhibition of the observed phenotype at the cellular and molecular level (Figure 5c and d), which further implicated TGFβ signaling in negatively regulating MSC differentiation in response to tumor CM. Thus, our findings corroborate previous studies suggesting a role for the TGFβ signaling pathway in regulating mesenchymal stem cell differentiation [31].

## Conclusions

Our data support an evolving hypothesis that cancer cells secrete a large number of factors regulating biological characteristics of MSCs and transforming MSCs into pro-inflammatory cells. We identified tumor-derived IL1β as one potential mediator of the observed phenotype. Nonetheless, we also identified FAK and MAPK signaling to regulate positively, while TGFβ signaling was found to negatively regulate the response of MSCs to tumor CM. Taken together, our data support a model where MSCs contribute to tumorigenicity through their pro-inflammatory phenotype induced by cancer cell-derived factors, such as IL1β (Figure 10b).

## Abbreviations

ALP: Alkaline phosphatase; BSA: Bovine serum albumin; CCL3: Chemokine (C-C motif) ligand 3; CCL5: Chemokine (C-C motif) ligand 5; CCL8: Chemokine (C-C motif) ligand 8; CM: Conditioned medium; CSF2: Colony stimulating factor 2; CSF3: Colony stimulating factor 3; CXCL1: Chemokine (C-X-C motif) ligand 1; CXCL2: Chemokine (C-X-C motif) ligand 2; CXCL3: Chemokine (C-X-C motif) ligand 3; CXCL5: Chemokine (C-X-C motif) ligand 5; CXCL6: Chemokine (C-X-C motif) ligand 6; (D)MEM: (D)ulbecco’s modified Eagle’s medium; DMSO: Dimethyl sulfoxide; EHF: Ets homologous factor; ELISA: Enzyme-linked immunosorbent assay; EMT: Epithelial mesenchymal transition; FAK: Focal adhesion kinase; FBS: Fetal bovine serum; GAPDH: Glyceraldehyde 3-phosphate dehydrogenase; H & E: Hematoxylin and eosin; hMSC-TERT-GFP: Human mesenchymal stem cell-telomerized-green fluorescence protein; IGF2: Insulin-like growth factor 2; IL13: Interleukin 13; IL1A: Interleukin 1, Alpha; IL1B: Interleukin 1, Beta; IL6: Interleukin 6; MAPK: Mitogen activated protein kinase; MMP12: Matrix metallopeptidase 12; MSCs: Mesenchymal stem cells; NEAA: Non-essential amino acids; PBMCs: Peripheral blood mononuclear cells; PBS: Phosphate-buffered saline; qRT-PCR: Quantitative real-time reverse-transcription PCR; SAA1: Serum amyloid A1; TGF-beta: Transforming growth factor beta; VEGF: Vascular endothelial growth factor A.

## Competing interests

The authors declare that they have no competing interests.

## Author’ contributions

M Al-toub performed the experiments and participated in preparing the manuscript; A Almusa, M Almajid performed the experiments; M Al-Nbaheen characterized MSCs phenotype; M Kassem, A Aldahamsh participated in study design, interpretation of data and preparation of the manuscript. M Kassem characterized and provided hMSC-TERT-GFP cell lines. NM Alajez was responsible for obtaining funding, study design, data interpretation, bioinformatics analysis and preparation of the manuscript. All authors read and approved the final manuscript.

## Supplementary Material

Additional file 1: Table S1 Pathway analysis of upregulated genes in MSCs exposed to FaDu CM. Click here for file

Additional file 2: Figure S1 Effect of FaDu CM on MSC cell growth and cell cycle. Click here for file

Additional file 3: Table S2 Functional annotation and clustering analysis of genes upregulated (10 fold) in MSCs exposed to FaDu CM. Click here for file

Additional file 4: Figure S2 Dose dependent effect of IL1β on MSC phenotype. Click here for file

Additional file 5: Figure S3 SB-431542 promotes the growth of MSCs in the presence of MDA-MB-231 CM. MSCs were grown in MDA-MB-231 CM in the presence of SB-431542 or DMSO. Cell viability was measured on days 3, 7, and 10 using alamar blue assay. Data are presented as mean ± S.D., n = 9. Click here for file

## References

[B1] Dominici M Le Blanc K Mueller I Slaper-Cortenbach I Marini F Krause D Deans R Keating A Prockop D Horwitz E Minimal criteria for defining multipotent mesenchymal stromal cells, The International Society for Cellular Therapy position statement Cytotherapy 2006 8 315 317 10.1080/14653240600855905 16923606

[B2] Granero-Molto F Weis JA Miga MI Landis B Myers TJ O’Rear L Longobardi L Jansen ED Mortlock DP Spagnoli A Regenerative effects of transplanted mesenchymal stem cells in fracture healing Stem Cells 2009 27 1887 1898 10.1002/stem.103 19544445 PMC3426453

[B3] Chen J Li Y Wang L Lu M Zhang X Chopp M Therapeutic benefit of intracerebral transplantation of bone marrow stromal cells after cerebral ischemia in rats J Neurol Sci 2001 189 49 57 10.1016/S0022-510X(01)00557-3 11535233

[B4] Wu GD Nolta JA Jin YS Barr ML Yu H Starnes VA Cramer DV Migration of mesenchymal stem cells to heart allografts during chronic rejection Transplantation 2003 75 679 685 10.1097/01.TP.0000048488.35010.95 12640309

[B5] Kristensen LP Chen L Nielsen MO Qanie DW Kratchmarova I Kassem M Andersen JS Temporal profiling and pulsed SILAC labeling identify novel secreted proteins during ex vivo osteoblast differentiation of human stromal stem cells Mol Cell Proteomics 2012 11 989 1007 10.1074/mcp.M111.012138 22801418 PMC3494153

[B6] Chamberlain G Fox J Ashton B Middleton J Concise review: mesenchymal stem cells: their phenotype, differentiation capacity, immunological features, and potential for homing Stem Cells 2007 25 2739 2749 10.1634/stemcells.2007-0197 17656645

[B7] Phinney DG Prockop DJ Concise review: mesenchymal stem/multipotent stromal cells: the state of transdifferentiation and modes of tissue repair–current views Stem Cells 2007 25 2896 2902 10.1634/stemcells.2007-0637 17901396

[B8] Kinnaird T Stabile E Burnett MS Lee CW Barr S Fuchs S Epstein SE Marrow-derived stromal cells express genes encoding a broad spectrum of arteriogenic cytokines and promote in vitro and in vivo arteriogenesis through paracrine mechanisms Circ Res 2004 94 678 685 10.1161/01.RES.0000118601.37875.AC 14739163

[B9] Karnoub AE Dash AB Vo AP Sullivan A Brooks MW Bell GW Richardson AL Polyak K Tubo R Weinberg RA Mesenchymal stem cells within tumour stroma promote breast cancer metastasis Nature 2007 449 557 563 10.1038/nature06188 17914389

[B10] Dwyer RM Potter-Beirne SM Harrington KA Lowery AJ Hennessy E Murphy JM Barry FP O’Brien T Kerin MJ Monocyte chemotactic protein-1 secreted by primary breast tumors stimulates migration of mesenchymal stem cells Clin Cancer Res 2007 13 5020 5027 10.1158/1078-0432.CCR-07-0731 17785552

[B11] Dvorak HF Tumors: wounds that do not heal. Similarities between tumor stroma generation and wound healing N Engl J Med 1986 315 1650 1659 10.1056/NEJM198612253152606 3537791

[B12] Erez N Truitt M Olson P Arron ST Hanahan D Cancer-associated fibroblasts are activated in incipient neoplasia to orchestrate tumor-promoting inflammation in an NF-kappaB-dependent manner Cancer Cell 2010 17 135 147 10.1016/j.ccr.2009.12.041 20138012

[B13] Navab R Strumpf D Bandarchi B Zhu CQ Pintilie M Ramnarine VR Ibrahimov E Radulovich N Leung L Barczyk M Panchal D To C Yun JJ Der S Shepherd FA Jurisica I Tsao MS Prognostic gene-expression signature of carcinoma-associated fibroblasts in non-small cell lung cancer Proc Natl Acad Sci U S A 2011 108 7160 7165 10.1073/pnas.1014506108 21474781 PMC3084093

[B14] Quante M Tu SP Tomita H Gonda T Wang SS Takashi S Baik GH Shibata W Diprete B Betz KS Friedman R Varro A Tycko B Wang TC Bone marrow-derived myofibroblasts contribute to the mesenchymal stem cell niche and promote tumor growth Cancer Cell 2011 19 257 272 10.1016/j.ccr.2011.01.020 21316604 PMC3060401

[B15] Lepperdinger G Brunauer R Jamnig A Laschober G Kassem M Controversial issue: is it safe to employ mesenchymal stem cells in cell-based therapies? Exp Gerontol 2008 43 1018 1023 10.1016/j.exger.2008.07.004 18694815

[B16] Khakoo AY Pati S Anderson SA Reid W Elshal MF Rovira II Nguyen AT Malide D Combs CA Hall G Zhang J Raffeld M Rogers TB Stetler-Stevenson W Frank JA Reitz M Finkel T Human mesenchymal stem cells exert potent antitumorigenic effects in a model of Kaposi’s sarcoma J Exp Med 2006 203 1235 1247 10.1084/jem.20051921 16636132 PMC2121206

[B17] Nakamizo A Marini F Amano T Khan A Studeny M Gumin J Chen J Hentschel S Vecil G Dembinski J Andreeff M Lang FF Human bone marrow-derived mesenchymal stem cells in the treatment of gliomas Cancer Res 2005 65 3307 3318 15833864 10.1158/0008-5472.CAN-04-1874

[B18] Zhu Y Sun Z Han Q Liao L Wang J Bian C Li J Yan X Liu Y Shao C Zhao RC Human mesenchymal stem cells inhibit cancer cell proliferation by secreting DKK-1 Leukemia 2009 23 925 933 10.1038/leu.2008.384 19148141

[B19] Shabahang M Buras RR Davoodi F Schumaker LM Nauta RJ Uskokovic MR Brenner RV Evans SR Growth inhibition of HT-29 human colon cancer cells by analogues of 1,25-dihydroxyvitamin D3 Cancer Res 1994 54 4057 4064 8033137

[B20] Alajez NM Shi W Wong D Lenarduzzi M Waldron J Weinreb I Liu FF Lin28b promotes head and neck cancer progression via modulation of the insulin-like growth factor survival pathway Oncotarget 2012 3 1641 1652 23482325 10.18632/oncotarget.785 PMC3681501

[B21] Shi W Gerster K Alajez NM Tsang J Waldron L Pintilie M Hui AB Sykes J P’ng C Miller N McCready D Fyles A Liu FF MicroRNA-301 mediates proliferation and invasion in human breast cancer Cancer Res 2011 71 2926 2937 10.1158/0008-5472.CAN-10-3369 21393507

[B22] Alajez NM Mocanu JD Krushel T Bell JC Liu FF Enhanced vesicular stomatitis virus (VSVDelta51) targeting of head and neck cancer in combination with radiation therapy or ZD6126 vascular disrupting agent Cancer Cell Int 2012 12 27 10.1186/1475-2867-12-27 22704542 PMC3487860

[B23] Banks-Schlegel SP Gazdar AF Harris CC Intermediate filament and cross-linked envelope expression in human lung tumor cell lines Cancer Res 1985 45 1187 1197 2578876

[B24] Simonsen JL Rosada C Serakinci N Justesen J Stenderup K Rattan SI Jensen TG Kassem M Telomerase expression extends the proliferative life-span and maintains the osteogenic potential of human bone marrow stromal cells Nat Biotechnol 2002 20 592 596 10.1038/nbt0602-592 12042863

[B25] Bentzon JF Stenderup K Hansen FD Schroder HD Abdallah BM Jensen TG Kassem M Tissue distribution and engraftment of human mesenchymal stem cells immortalized by human telomerase reverse transcriptase gene Biochem Biophys Res Commun 2005 330 633 640 10.1016/j.bbrc.2005.03.072 15809044

[B26] Alajez NM Shi W Hui AB Yue S Ng R Lo KW Bastianutto C O’Sullivan B Gullane P Liu FF Targeted depletion of BMI1 sensitizes tumor cells to P53-mediated apoptosis in response to radiation therapy Cell Death Differ 2009 16 1469 1479 10.1038/cdd.2009.85 19575017

[B27] Alajez N Shi W Hui A Bruce J Lenarduzzi M Ito E Yue S O’Sullivan B Liu F Enhancer of Zeste homolog 2 (EZH2) is overexpressed in recurrent nasopharyngeal carcinoma and is regulated by miR-26a, miR-101, and miR-98 Cell Death Dis 2010 1 e85 10.1038/cddis.2010.64 21368858 PMC3035896

[B28] Vishnubalaji R Manikandan M Al-Nbaheen M Kadalmani B Aldahmash A Alajez NM In vitro differentiation of human skin-derived multipotent stromal cells into putative endothelial-like cells BMC Dev Biol 2012 12 7 10.1186/1471-213X-12-7 22280443 PMC3280173

[B29] Grunert S Jechlinger M Beug H Diverse cellular and molecular mechanisms contribute to epithelial plasticity and metastasis Nat Rev Mol Cell Biol 2003 4 657 665 10.1038/nrm1175 12923528

[B30] Ganapathy V Ge R Grazioli A Xie W Banach-Petrosky W Kang Y Lonning S McPherson J Yingling JM Biswas S Mundy GR Reiss M Targeting the Transforming Growth Factor-beta pathway inhibits human basal-like breast cancer metastasis Mol Cancer 2010 9 122 10.1186/1476-4598-9-122 20504320 PMC2890606

[B31] Roelen BA Dijke P Controlling mesenchymal stem cell differentiation by TGFBeta family members J Orthop Sci 2003 8 740 748 10.1007/s00776-003-0702-2 14557946

[B32] Birnbaum T Hildebrandt J Nuebling G Sostak P Straube A Glioblastoma-dependent differentiation and angiogenic potential of human mesenchymal stem cells in vitro J Neurooncol 2011 105 57 65 10.1007/s11060-011-0561-1 21547397

[B33] Gene Expression Omnibushttp://www.ncbi.nlm.nih.gov/geo

[B34] Grivennikov SI Greten FR Karin M Immunity, inflammation, and cancer Cell 2010 140 883 899 10.1016/j.cell.2010.01.025 20303878 PMC2866629

[B35] Kuraishy A Karin M Grivennikov SI Tumor promotion via injury- and death-induced inflammation Immunity 2011 35 467 477 10.1016/j.immuni.2011.09.006 22035839 PMC3587290

[B36] Albini A Sporn MB The tumour microenvironment as a target for chemoprevention Nat Rev Cancer 2007 7 139 147 17218951 10.1038/nrc2067

[B37] Chaffer CL Weinberg RA A perspective on cancer cell metastasis Science 2011 331 1559 1564 10.1126/science.1203543 21436443

[B38] Liu S Ginestier C Ou SJ Clouthier SG Patel SH Monville F Korkaya H Heath A Dutcher J Kleer CG Jung Y Dontu G Taichman R Wicha MS Breast cancer stem cells are regulated by mesenchymal stem cells through cytokine networks Cancer Res 2011 71 614 624 10.1158/0008-5472.CAN-10-0538 21224357 PMC3100554

[B39] Goldstein RH Reagan MR Anderson K Kaplan DL Rosenblatt M Human bone marrow-derived MSCs can home to orthotopic breast cancer tumors and promote bone metastasis Cancer Res 2010 70 10044 10050 10.1158/0008-5472.CAN-10-1254 21159629 PMC3017423

[B40] Studeny M Marini FC Dembinski JL Zompetta C Cabreira-Hansen M Bekele BN Champlin RE Andreeff M Mesenchymal stem cells: potential precursors for tumor stroma and targeted-delivery vehicles for anticancer agents J Natl Cancer Inst 2004 96 1593 1603 10.1093/jnci/djh299 15523088

[B41] Loebinger MR Kyrtatos PG Turmaine M Price AN Pankhurst Q Lythgoe MF Janes SM Magnetic resonance imaging of mesenchymal stem cells homing to pulmonary metastases using biocompatible magnetic nanoparticles Cancer Res 2009 69 8862 8867 10.1158/0008-5472.CAN-09-1912 19920196 PMC2833408

[B42] Komarova S Roth J Alvarez R Curiel DT Pereboeva L Targeting of mesenchymal stem cells to ovarian tumors via an artificial receptor J Ovarian Res 2010 3 12 10.1186/1757-2215-3-12 20500878 PMC2883983

[B43] Djouad F Plence P Bony C Tropel P Apparailly F Sany J Noel D Jorgensen C Immunosuppressive effect of mesenchymal stem cells favors tumor growth in allogeneic animals Blood 2003 102 3837 3844 10.1182/blood-2003-04-1193 12881305

[B44] Brune JC Tormin A Johansson MC Rissler P Brosjo O Lofvenberg R von Steyern FV Mertens F Rydholm A Scheding S Mesenchymal stromal cells from primary osteosarcoma are non-malignant and strikingly similar to their bone marrow counterparts Int J Cancer 2011 129 319 330 10.1002/ijc.25697 20878957

[B45] Hung SC Deng WP Yang WK Liu RS Lee CC Su TC Lin RJ Yang DM Chang CW Chen WH Wei HJ Gelovani JG Mesenchymal stem cell targeting of microscopic tumors and tumor stroma development monitored by noninvasive in vivo positron emission tomography imaging Clin Cancer Res 2005 11 7749 7756 10.1158/1078-0432.CCR-05-0876 16278396

[B46] McGrail DJ Ghosh D Quach ND Dawson MR Differential mechanical response of mesenchymal stem cells and fibroblasts to tumor-secreted soluble factors PLoS One 2012 7 e33248 10.1371/journal.pone.0033248 22438903 PMC3306382

[B47] Acharyya S Oskarsson T Vanharanta S Malladi S Kim J Morris PG Manova-Todorova K Leversha M Hogg N Seshan VE Norton L Brogi E Massagué J A CXCL1 paracrine network links cancer chemoresistance and metastasis Cell 2012 150 165 178 10.1016/j.cell.2012.04.042 22770218 PMC3528019

[B48] Oue E Lee JW Sakamoto K Iimura T Aoki K Kayamori K Michi Y Yamashiro M Harada K Amagasa T Yamaguchi A CXCL2 synthesized by oral squamous cell carcinoma is involved in cancer-associated bone destruction Biochem Biophys Res Commun 2012 424 456 461 10.1016/j.bbrc.2012.06.132 22771802

[B49] Burns JS Kristiansen M Kristensen LP Larsen KH Nielsen MO Christiansen H Nehlin J Andersen JS Kassem M Decellularized matrix from tumorigenic human mesenchymal stem cells promotes neovascularization with galectin-1 dependent endothelial interaction PLoS One 2011 6 e21888 10.1371/journal.pone.0021888 21779348 PMC3133605

[B50] Robles Irizarry L Hambardzumyan D Nakano I Gladson CL Ahluwalia MS Therapeutic targeting of VEGF in the treatment of glioblastoma Expert Opin Ther Targets 2012 16 973 984 10.1517/14728222.2012.711817 22876981

