# Supplementary material for: Pleiotropic effects of cancer cells’ secreted factors on human stromal (mesenchymal) stem cells
Source: Stem Cell Res Ther. 2013 Sep 17;4(5):114. doi: 10.1186/scrt325 (PMC3854757; doi:10.1186/scrt325)
Supplement: Additional file 2: Figure S1 — Effect of FaDu CM on MSC cell growth and cell cycle. [file scrt325-S2.pdf]

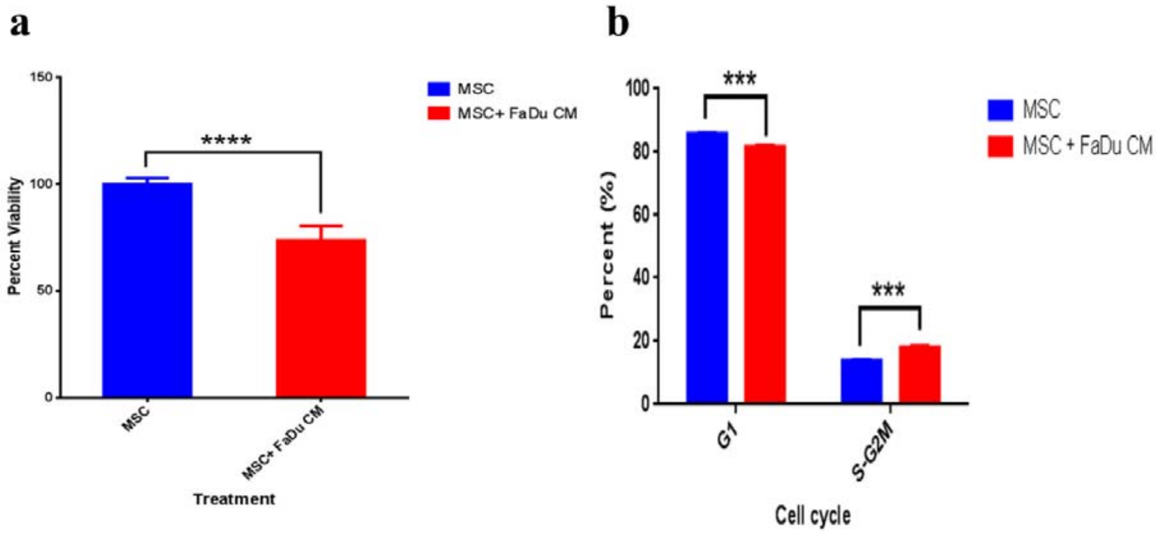

Supplementary figure 1. Effect of FaDu CM on MSC cell growth and cell cycle. (a) MSCs were cultured in normal DMEM or in FaDu CM and on day 4, cell viability was measured using MTT assay. Data are presented as mean ± S.D., n=5. **(b)** MSCs were cultured as in (a), then cell cycle analysis was done on control MSCs or MSCs cultured in FaDu CM on day 5. Data are presented as mean ± S.D., n=3.
