# Supplementary material for: Pleiotropic effects of cancer cells’ secreted factors on human stromal (mesenchymal) stem cells
Source: Stem Cell Res Ther. 2013 Sep 17;4(5):114. doi: 10.1186/scrt325 (PMC3854757; doi:10.1186/scrt325)
Supplement: Additional file 4: Figure S2 — Dose dependent effect of IL1β on MSC phenotype. [file scrt325-S4.pdf]

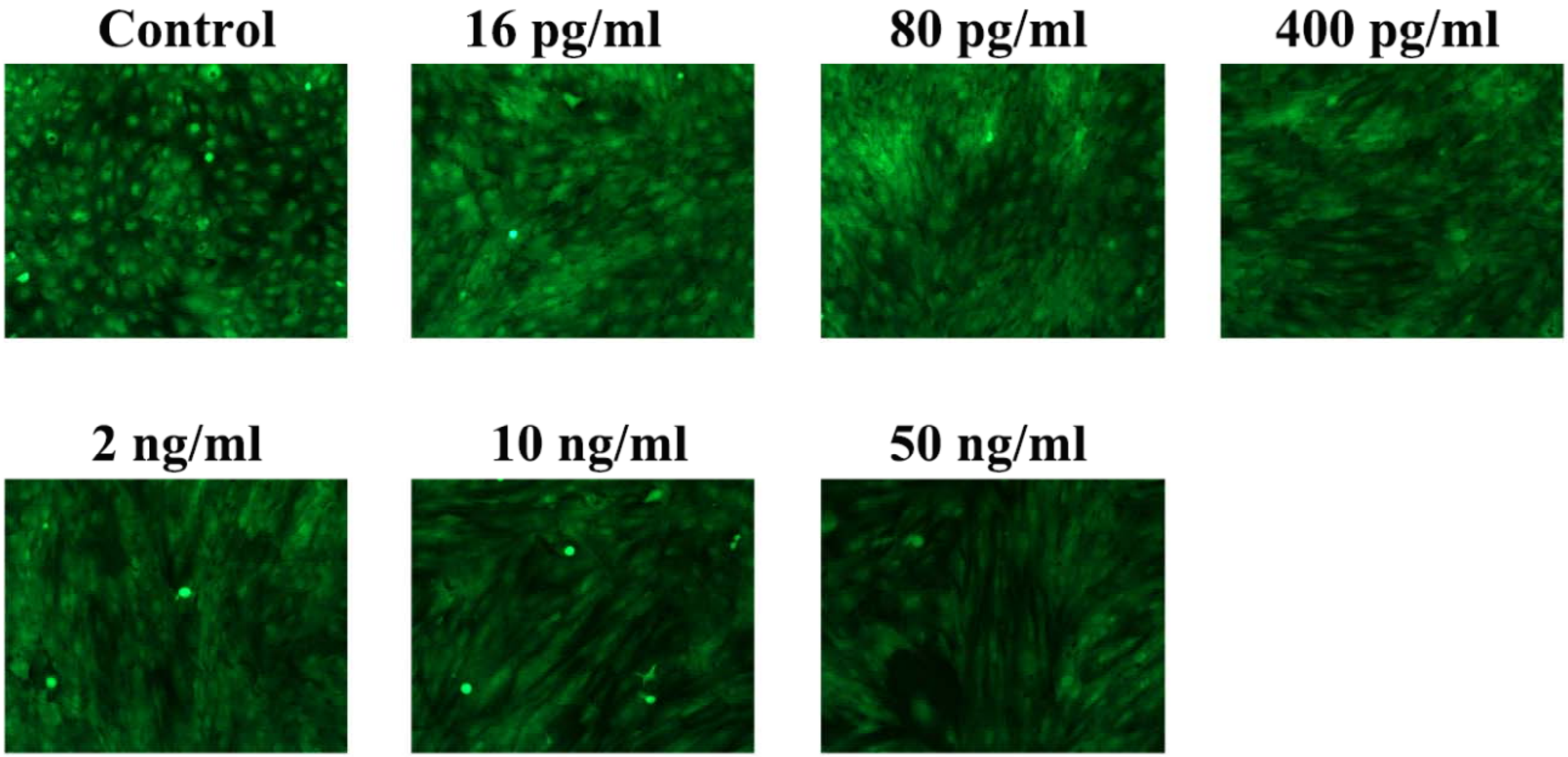

Supplementary figure 2. Dose dependent effect of IL1β on MSC phenotype. MSCs were treated with the indicated dose of IL1β and changes in cell morphology were monitored on day 7. Images were taken at 10x magnification.
