# Supplementary material for: Pleiotropic effects of cancer cells’ secreted factors on human stromal (mesenchymal) stem cells
Source: Stem Cell Res Ther. 2013 Sep 17;4(5):114. doi: 10.1186/scrt325 (PMC3854757; doi:10.1186/scrt325)
Supplement: Additional file 5: Figure S3 — SB-431542 promotes the growth of MSCs in the presence of MDA-MB-231 CM. MSCs were grown in MDA-MB-231 CM in the presence of SB-431542 or DMSO. Cell viability was measured on days 3, 7, and 10 using alamar blue assay. Data are presented as mean ± S.D., n = 9. [file scrt325-S5.pdf]

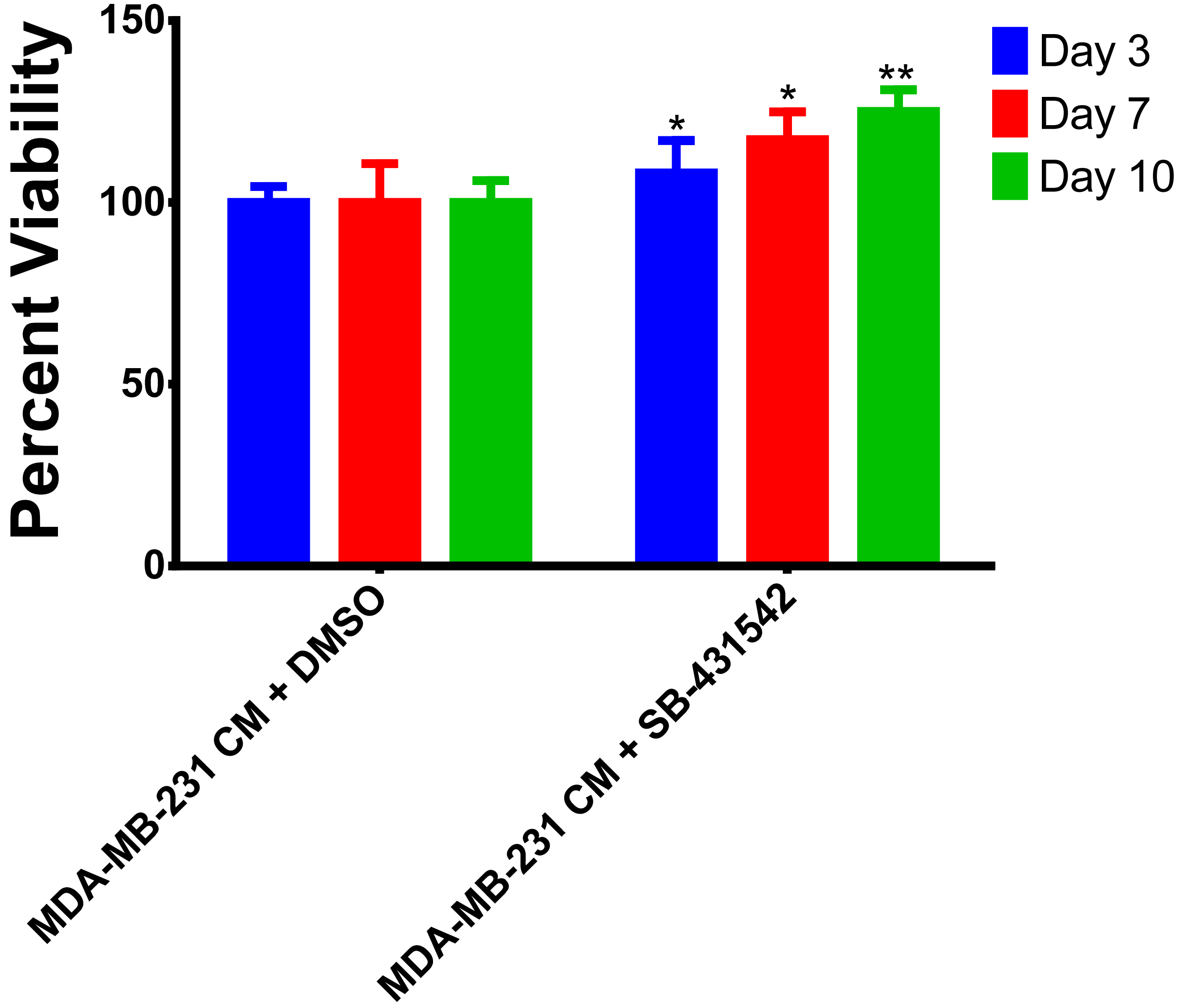

Supplementary Figure 3. SB-431542 promotes the growth of MSCs in the presence of MDA-MB-231 CM. MSCs were grown in MDA-MB -231 CM in MDA-MB-231 CM the presence of SB-431542 or DMSO. Cell viability was measured on days 3, 7, and 10 using alarm blue assay. Data are presented as mean ± S.D., n=9.
